# Automated high-throughput screening combined with model-assisted analysis enables quantitative identification of process drivers and trade-offs during extracellular Fab production in *Escherichia coli*

**DOI:** 10.1007/s00449-026-03379-7

**Published:** 2026-08-14

**Authors:** Fabian Schröder-Kleeberg, Lucas Kaspersetz, Sanchir Anar Neff, Markus Zoellkau, Markus Glaser, Markus Brunner, Christian Bosch, Mariano Nicolas Cruz Bournazou, Peter Neubauer

**Affiliations:** 1 Department of Bioprocess Engineering, Institute of Biotechnology, Technische Universität Berlin, Ackerstr. 76, 13355 Berlin, Germany; 2 Wacker Biotech GmbH, 07745 Jena, Germany; 3 Wacker Chemie AG, 81379 München, Germany

**Keywords:** High-throughput bioprocess development, Fed-batch cultivation, Mechanistic modeling, Fab antibody production, Leaky *Escherichia coli*, Process optimization

## Abstract

Production of Fab antibody fragments with *Escherichia coli* strains engineered for extracellular product release can reduce downstream processing requirements but often compromises cellular integrity, resulting in increased cell lysis. To investigate the balance between product formation, product release, and cellular robustness, 32 fed-batch cultivation conditions spanning six process parameters were systematically evaluated in automated high-throughput bioreactors. In total, 100 cultivations were performed, and mechanistic modeling was applied to estimate cell lysis, product formation, and product release kinetics. The combined experimental and model-based analysis revealed a pronounced trade-off between productivity and robustness. Conditions yielding high Fab titers showed minimal cell lysis rates but also slow product release, whereas elevated release kinetics were consistently associated with substantial lysis and reduced Fab titers. Induction strength emerged as the primary driver for maximizing Fab titer, while production temperature governed the balance between product release and cell lysis. Compared to the reference condition, Fab titers increased approximately twofold to 730 mg L$^{-1}$ while reducing the cell lysis rate by up to eightfold to near-negligible levels, albeit at the expense of a 2.2-fold lower product release rate. Robustness studies under large-scale–relevant conditions identified moderate substrate heterogeneities as an additional factor enhancing productivity while reducing cell lysis. Furthermore, the extensive dataset enabled refinement of a previously developed product model by introducing an induction-strength-dependent production load and provided mechanistic insight into the relationship between product release and cell lysis. Overall, the study demonstrates how mechanistic modeling enhances the interpretation of automated high-throughput screening experiments by linking observable process responses to underlying physiological behavior and enabling quantitative analysis of complex production trade-offs.

## Introduction

Recombinant antibody fragments such as Fabs and scFvs do not require post-translational glycosylation, making *Escherichia coli* an attractive production host. Fabs can be produced in *E. coli* either (i) in inclusion bodies (IBs) followed by refolding or (ii) as soluble fragments. The latter strategy relies on Sec-dependent translocation in the periplasm, where correct folding and disulfide bond formation occur [1]. Helper proteins, including disulfide isomerase [2] and the periplasmic chaperone Skp [3], can substantially enhance the expression yields. Alternatively, cytoplasmic disulfide bond formation systems (CyDisCo) allow Fab production in the cytoplasm [4] and subsequent transport to the periplasm via the Tat pathway [5].

To release fully functional periplasmic products into the reactor medium, engineering the outer membrane to increase permeability or leakage enables passive transport [1, 6]. However, this approach often destabilizes the membrane, increasing the risk of cell lysis under suboptimal conditions [7, 8]. Numerous studies have sought to improve the release of products using molecular biology strategies, including signal peptide engineering [9, 10], gene deletions that improve permeability [11], or induced downregulation of key genes [12]. Chemical additives such as EDTA, Triton X-100, and glycine have also beenexplored [13].

An alternative and complementary strategy is the optimization of cultivation conditions. Key parameters include temperature [7, 8, 14, 15], pre-induction growth rate ($\mu _{\text {set}})$ [16, 17], feeding profiles during production [7, 8], aeration [18], and inducer type and strength [19]. However, these studies often focus on individual process parameters and therefore provide limited insight into the interactions and trade-offs governing extracellular Fab production under fed-batch conditions.

To address this gap, we investigated the effects and interactions of production temperature, pre-induction growth rate, induction strength, and post-induction feed rate in a Fab production process in *E. coli* employing a two-step secretion mechanism comprising Sec-dependent translocation into the periplasm followed by passive product release enabled by modifications in the outer membrane, similar to the strain described by Leonhartsberger [20]. In addition, the influence of initial yeast extract concentration in the batch medium and heterogeneous substrate conditions relevant to large-scale operation was investigated to assess process robustness beyond standard fed-batch operation.

Our evaluation focused on process-controllable criteria that capture the central trade-off of extracellular Fab production: maximizing product formation and extracellular product accumulation while maintaining cellular robustness. Therefore, product titer and cellular robustness were selected as primary process performance criteria. Cellular robustness, product formation kinetics, and product release kinetics were obtained through model-based estimation of physiological parameters, whereas product titer was directly determined experimentally. The simplicity and robustness of this modeling approach have previously been demonstrated in strain characterization [21, 22] and scale-down studies [23–25].

This study demonstrates the advantages of integrating automated high-throughput screening with model-assisted analysis for bioprocess optimization. The screening campaign enabled identification of process conditions that improved Fab production and cellular robustness, while the model-assisted analysis provided mechanistic insight into the underlying trade-off between product release and cell lysis. Production temperature and induction strength emerged as the dominant process drivers shaping this trade-off. Together, these results illustrate how combining high-throughput experimentation with physiological modeling can support more knowledge-driven process development.

## Material and methods

### Strain, media and preculture

The screening experiments were performed with a genetically modified *E. coli* K-12 (tet^R^; *lacY*^*+*^) precursor secretion strain kindly provided by Wacker Chemie AG. The strain carried a pMT2 plasmid encoding a Fab antibody fragment inducible by isopropyl-*β*-D-thiogalactopyranoside (IPTG) and contained mutations in genes leading to altered outer membrane permeability to enhance product release into the medium.

A mineral salt medium (MSM), as based on [26], was used for both pre- and main cultures. Yeast extract was added to the MSM, with identical concentrations in the preculture and batch medium; additional details on yeast extract levels are provided below.

The preculture was supplemented with 10 g L$$^{-1}$$ EnPump200 glucose polymer and 8 U L$$^{-1}$$ Reagent A (Enpresso GmbH, Berlin, Germany), 5 g L$$^{-1}$$ glucose, antifoam (1:10 000 dilution), and 20 mg L$$^{-1}$$ tetracycline. It was inoculated to an optical density at 600 nm (OD$$_{600}) $$ of 0.02 from a cryoculture in a disposable 125 mL Erlenmeyer flask with baffles and incubated at 30 $$^\circ $$C and 200 rpm in an orbital shaker with 50 mm amplitude for up to 15 h overnight (Adolf Kühner AG, Birsfelden, Switzerland).

The shake flasks were equipped with optical sensor spots for online monitoring of dissolved oxygen tension (DOT) and pH on a PreSens platform (PreSens–Precision Sensing GmbH, Regensburg, Germany). Online monitoring was used to ensure that the precultures were not exposed to oxygen limitation or saturation, thereby promoting consistent physiological starting conditions for each cultivation.

### Cultivation systems and default cultivation conditions

The screening experiments were performed in the automated KIWI-biolab of the Technical University of Berlin, which is equipped with a bioREACTOR48^®^ (2mag AG, München, Germany). The bioREACTOR48^®^ accommodates up to 48 single-use 15 mL mini-bioreactors (MBRs) operated in parallel. The working volume used in this study was 10 mL. Each MBR is equipped with optical sensor spots (PreSens–Precision Sensing GmbH, Regensburg, Germany) for monitoring pH and dissolved oxygen tension (DOT). External controls including sampling, feeding, pH regulation, and volume control were fully automated via a Tecan Liquid Handling Station (LHS) (Tecan Group Ltd., Männedorf, Switzerland). The magnetically driven impeller was operated at a constant stirring speed of 2800 rpm. The reactor block was aerated with 5 L$_\text {n}$ min$^{-1}$ gas flow enriched with 30% oxygen. For details on the design, workflow management, measurement principles, and additional control options of the small-scale high-throughput facility, the reader is referred to [27, 28].

Fed-batch cultivations with continuous feeding, as well as pulse-based feeding for reference and validation purposes, were performed in the BioXplorer100 (H.E.L Group, London, United Kingdom), which enables parallel operation of six 150 mL stirred-tank reactors. The working volume of each reactor was 85 mL. Feeding and pH control were executed via pump-driven control units. In pulse-based feeding experiments, a single pulse corresponded to a 30 s pump activation. To ensure comparable substrate input between continuous and pulse-based feeding during the fed-batch phase, each pulse delivered the equivalent amount of substrate supplied by continuous feeding over the respective pulse interval. Each reactor was equipped with pH and DOT sensors (AppliSens, Applikon Biotechnology B.V., Delft, Netherlands). Reactor temperature was controlled by a PolyBlock heating/cooling unit (H.E.L Group) supported by a Minichiller (Peter Huber Kältemaschinenbau SE, Offenburg, Germany), which allowed cooling to 4 $^\circ$C for low-temperature processes.

The system was aerated with compressed air at 40 mL$_\text {n}$ min$^{-1}$ during the batch phase and 150 mL$_\text {n}$ min$^{-1}$ during the fed-batch phase. Stirring was increased from 1000 rpm during the batch phase to 1500 rpm during the fed-batch phase. Integration of the reactors with a Tecan LHS and a mobile robotic arm enabled complete automation up to at-line analytics. Additional information on system setup, measurement principles, workflow, and data management is provided in [29, 30].

In both cultivation systems, the pH was one-sided controlled at pH 6.8 using 7.5% NH$$_3 , $$ and the initial cultivation temperature was set to 30 $$^\circ $$C. At the end of the batch phase, the exponential feed rate $$F_{\text {exp}}$$ (L h$$^{-1}) $$ was calculated from the initial feed rate $$F_{0} , $$ the applied specific growth rate $$\mu _{\text {set}}$$ (h$$^{-1}) $$, and the time *t* (h):

1 $$\begin{aligned} F_{\text {exp}}(t) = F_{0} \, e^{\mu _{\text {set}} t} \end{aligned}$$

The initial feed rate $$F_{0}$$ was derived from $$\mu _{\text {set}} , $$ the glucose concentration in the feed $$S_{i}$$ (g L$$^{-1}) $$, the biomass yield coefficient $$Y_{X/S} , $$ the starting volume $$V_{0} , $$ and the biomass concentration at the onset of feeding in each MBR, $$X_{0}$$:

2 $$\begin{aligned} F_{0} = \frac{\mu _{\text {set}}}{S_{i} \, Y_{X/S}} \, X_{0} \, V_{0} \end{aligned}$$

The biomass yield coefficient $Y_{X/S} = 0.34\,\mathrm {g\,g^{-1}}$ was derived from experimental data obtained in Schröder-Kleeberg et al. [25]. Across all experiments, a induction biomass concentration ($X_{\text {induction}})$ of $\text {OD}_{600} = 50$ was targeted at the time of induction. Consequently, the duration of the exponential growth phase had to be adjusted according $\mu _{\text {set}}$ and estimated using equation (3):

3 $$\begin{aligned} \mu _{\text {set}} = \frac{\ln \!\left( X_{\text {induction}}\right) - \ln \!\left( X_{\text {0}}\right) }{\Delta t} \end{aligned}$$

### Experimental approach

The bioprocess was divided into three phases: (1) a batch phase, (2) a non-induced exponentially fed growth phase, and (3) an induced and constantly fed production phase. In each phase, at least one specific process parameter was varied to investigate its effect on Fab titer, cell lysis rate ($\textrm{q}_{\textrm{d}})$, the specific product formation rate ($\textrm{q}_{\textrm{P}})$, and the specific product release into the medium ($\textrm{q}_{\textrm{P,release}})$.

**Table 1:** Experimental design

Condition	Replicates	$F_{\mathrm {\tau }}\,[\textrm{min}]$	$\mu _{\textrm{set}}\,[\textrm{h}^{-1}]$	$I_{\textrm{IPTG}}\,[\mu \textrm{M}]$	$T_{\textrm{prod}}\,[^\circ \textrm{C}]$	F_X,prod_ [g g^−1^ h^−1^]	YE_init_ [g L^−1^]
C1 (Reference)	8	5	stepwise	100	30	0.09	10
C2	8	10	stepwise	100	30	0.09	10
C3	3	5	0.15	100	30	0.18	7.5
C4	3	5	0.15	100	30	0.36	7.5
C5	3	5	0.2	50	30	0.18	7.5
C6	3	5	0.2	100	30	0.18	7.5
C7	3	5	0.2	100	30	0.36	7.5
C8	3	5	0.2	200	30	0.18	7.5
C9	3	5	0.3	100	30	0.18	7.5
C10	3	5	0.3	100	30	0.36	7.5
C11	2	5	0.2	25	25	0.09	5
C12	3	5	0.2	25	25	0.18	5
C13	3	5	0.2	25	25	0.36	5
C14	2	5	0.2	50	25	0.09	5
C15	3	5	0.2	50	25	0.18	5
C16	3	5	0.2	50	25	0.36	5
C17	2	5	0.2	100	25	0.09	5
C18	3	5	0.2	100	25	0.18	5
C19	3	5	0.2	100	25	0.36	5
C20	3	0	0.2	50	25	0.09	7.5
C21 (Validation)	3	5	0.2	50	25	0.09	7.5
C22	2	5	0.2	10	25	0.09	5
C23	3	5	0.2	10	25	0.18	5
C24	3	5	0.2	10	25	0.36	5
C25	3	5	0.2	2	25	0.18	7.5
C26	2	5	0.2	2	25	0.36	7.5
C27	3	5	0.2	10	25	0.09	7.5
C28	3	5	0.2	10	25	0.18	7.5
C29	3	5	0.2	10	25	0.36	7.5
C30	3	5	0.2	50	25	0.09	7.5
C31	3	5	0.2	50	25	0.18	7.5
C32	2	5	0.2	50	25	0.36	7.5

The investigated process parameters were the initial yeast extract concentration ($\textit{YE}_{\textrm{init}})$, the post-induction specific feeding rate normalized to the induction biomass ($F_{\textrm{X,prod}})$, the pre-induction specific growth rate ($\mu _{\textrm{set}})$, the production temperature ($T_{\textrm{prod}})$, the induction strength ($I_{\textrm{IPTG}})$, and the feeding regime ($F_{\mathrm {\tau }})$. $F_{\mathrm {\tau }}$ denotes the feeding interval, where a value of 0 corresponds to continuous feeding and values greater than 0 correspond to pulse-based feeding

In the batch phase, the factor varied was the initial yeast extract concentration ($$\textit{YE}_{\textrm{init}}) $$ in the range of 5, 7.5, or 10 g L$$^{-1} . $$ Fed-batch operation was initiated after depletion of the initial glucose concentration in the batch phase, with a specific pre-induction growth rate ($$\mu _{\textrm{set}}) $$ of 0.15, 0.2, or 0.3 h$$^{-1} . $$

After induction, the following factors were varied: induction strength with IPTG ($$I_{\textrm{IPTG}}) $$ at 2, 10, 25, 50, 100, or 200 *μ *M; production temperature ($$T_{\textrm{prod}}) $$ at 25 or 30 $$^\circ $$C; and the specific post-induction feeding rate normalized to induction biomass ($$F_{\textrm{X,prod}}) $$ at 0.09, 0.18, or 0.36 g g$$^{-1}$$ h$$^{-1} . $$

The final investigated factor was the feeding regime ($F_{\mathrm {\tau }})$, evaluated as continuous feeding ($F_{\mathrm {\tau }}= 0$ min) or pulse-based feeding with pulse intervals of 5 or 10 min. In the BioXplorer100 system, comparisons between continuous feeding and a 5 min pulsed feeding regime were performed. As the bioREACTOR48^®^ only supports pulse-based feeding, the influence of pulse frequency was assessed during the initial screening phase by comparing pulse intervals of 5 and 10 min. In addition, the BioXplorer100 experiments were used to validate one of the best-performing conditions identified in the optimization study, as this system provided improved oxygen control. In total, these variations resulted in 32 combinations, most of which were performed in triplicate (Table 1).

The initial conditions, referred to as the reference condition for the optimization screening, were: $$T_{\textrm{prod}}= 30~^\circ $$C, $$F_{\textrm{X,prod}}= 0.09$$ g g$$^{-1}$$ h$$^{-1} , $$ $$I_{\textrm{IPTG}}= 100~\mu $$M, $$F_{\mathrm {\tau }}= 5$$ min, and an initial yeast extract concentration of 10 g L$$^{-1} . $$ A stepwise feeding profile was applied, resulting in dynamic changes of $$\mu _{\textrm{set}}$$ over time [31].

The experimental design listed in Table 1 was structured as follows: the production temperature ($T_{\textrm{prod}})$ was treated as a qualitative factor. At $T_{\textrm{prod}}= 30~^\circ$C, the effects of$\mu _{\textrm{set}},$ $I_{\textrm{IPTG}},$ and $F_{\textrm{X,prod}}$ were evaluated in a central composite face-centered (CCF) design, including robustness testing for $F_{\textrm{X,prod}} .$ At a production temperature of 25 $^\circ$C, $I_{\textrm{IPTG}}$ and $F_{\textrm{X,prod}}$ were evaluated using a full factorial design, while the comparison between continuous and pulse-based feeding ($F_{\mathrm {\tau }})$ was investigated using a fractional factorial design. The impact of larger inhomogeneities (5–10 min) was examined independently of this comparison at $T_{\textrm{prod}}= 30~^\circ$C (Table 1, C1 and C2). All time-resolved data for biomass, glucose, acetate, total product, extracellular product, base addition, DOT, and pH are presented in Figs. S1–S12 (Supplementary Information 1).

### Analytics

Online pH and DOT measurements were performed as described above. For at-line measurements in the bioREACTOR48^®^ and BioXplorer100 cultivations, samples were transferred into a pre-chilled 96-well microplate containing dried NaOH, corresponding to 15 *μ *L of 2 M NaOH per well. Upon sample addition, the NaOH dissolved and increased the pH, thereby inhibiting cell activity without causing cell lysis in *E. coli* [32].

The OD$_{600}$ was measured using a Microlab^®^ STAR liquid-handling system (Hamilton Company, Bonaduz, Switzerland) equipped with an integrated Synergy MX microplate reader (BioTek Instruments GmbH, Bad Friedrichshall, Germany). For modeling purposes, OD$_{600}$ values were converted to dry cell weight using a strain-specific conversion factor of $0.37~\mathrm {g\,L^{-1}}$ per OD$_{600}$ unit, previously established using a spectrophotometer (Amersham Biosciences, Amersham, United Kingdom). Since OD$_{600}$ values obtained with the microplate reader differed systematically from spectrophotometer measurements, an experimentally determined calibration factor of 2.7 was applied to the microplate reader data prior to biomass conversion.

Cells were separated from the supernatant by centrifugation at 15,000 *g* for 10 min at 4 $^\circ$C. Glucose and acetate concentrations in the supernatant were quantified using a Cedex Bio HT Analyzer (I&L Biosystems GmbH, Königswinter, Germany). Extracellular and total Fab antibody concentrations were determined via sandwich ELISA. Each sample was measured in technical quadruplicates. Identical sample preparation, dilution, calibration, and assay procedures were applied across all cultivations. Samples were analyzed at high dilution factors (typically 1:40,000–1:70,000), thereby reducing the potential influence of intracellular matrix components released during cell lysis. The complete protocol for product quantification is provided in Schröder-Kleeberg et al. [25].

### Mechanistic model

The mechanistic model underlying this study was originally developed by Schröder-Kleeberg et al. [25]. The complete set of model equations and nomenclature is provided in Appendix A.1 and Appendix A.2, respectively.

The model describes the growth kinetics of *E. coli* cultivated in a semi-defined medium containing a defined limiting substrate (*S*) and yeast extract ($\textrm{YE})$ as a complex additive. In this framework, yeast extract is partitioned into two subfractions ($\mathrm {YEF_A}$ and $\mathrm {YEF_B})$, representing rapidly and slowly utilized fractions with distinct physiological effects on *E. coli* metabolism.

To describe product formation $( \textrm{q}_{\textrm{P}}) ,$ the model distinguishes between Fab fragments located in the periplasm $(P_{\textrm{Fab,PP}})$ and Fab fragments released into the reactor medium $(P_{\textrm{Fab,M}}) .$ Product release was described via the specific product release rate $(\textrm{q}_{\textrm{P,release}}) ,$ representing the transport of Fab fragments across the outer membrane into the cultivation broth.

The production strain used in this study was based on an outer membrane engineering approach enabling extracellular Fab release. While product release itself is distinct from cell lysis, the membrane modifications increased membrane fragility and thereby susceptibility to lysis following induction. Consequently, extracellular product accumulation may result either from the engineered passive release mechanism, lysis-associated release, or a combination of both processes.

To account for the observed biomass decline during cultivation, a functional cell lysis parameter $(\textrm{q}_{\textrm{d}})$ was introduced and estimated from macroscopic biomass balances. The parameter represents an additional sink term required to describe biomass loss not explained by growth and product formation and was used as a comparative indicator of process-dependent effects on cell stability.

In the present study, the representation of induction behavior was adapted. Induction was assumed to impose a production load $(p_{\textrm{load}})$ that is limited by the maximum oxidative substrate flux capacity, represented by $\hat{q}_{S,\textrm{ox,max}} .$ Consequently, the product-related oxidative substrate flux $q_{S,\textrm{ox},P}$ from the original model formulation (Eq. 31) was modified as follows:

4 $$\begin{aligned} q_{S,\textrm{ox},P} = {\left\{ \begin{array}{ll} \hat{q}_{S,\textrm{ox,max}} \, p_{\textrm{load}} \, e^{-P/K_{P}}, \\ q_{S,\textrm{ox}}, & \text {otherwise}, \end{array}\right. } \end{aligned}$$

where $\hat{q}_{S,\textrm{ox,max}}$ is estimated according to:

5 $$\begin{aligned} \hat{q}_{S,\textrm{ox,max}} = q_{S,\textrm{max}} \, \textrm{TIF} \, \frac{K_{qS\textrm{ox}}}{K_{qS\textrm{ox}} + q_{S,\textrm{max}} \, \textrm{TIF}}. \end{aligned}$$

Since product formation is not represented explicitly as a model state variable, the maximum specific product formation rate was estimated as:

6 $$\begin{aligned} \hat{q}_{P,\max } = \hat{q}_{S,\textrm{ox,max}} \, p_{\textrm{load}} \, Y_{P/S}. \end{aligned}$$

To distinguish between lysis-associated and viable biomass-associated product release, the model was extended by introducing separate release terms. The specific lysis-associated product release rate was described as:

7 $$\begin{aligned} q_{P,\textrm{rL}} = q_{d}\, \frac{P_{\textrm{Fab,PP}}}{X}, \end{aligned}$$

where the release of product is directly coupled to the cell lysis rate and depends on the amount of product present in the periplasm per biomass.

Accordingly, the amount of product released by lysis was described as

8 $$\begin{aligned} \frac{dP_{\textrm{Fab,Lysis}}}{dt} = - \frac{F}{V} \, P_{\textrm{Fab,Lysis}} + q_{d} \, P_{\textrm{Fab,PP}}. \end{aligned}$$

The viable biomass-associated specific product release rate replaced the original model formulation (Eq. 33) and was described by $q_{P,\textrm{rX}}$:

9 $$\begin{aligned} q_{P,\textrm{rX}} = q_{\textrm{P,release,max}} \, \frac{P_{\textrm{Fab,PP}}}{P_{\textrm{Fab,PP}} + K_{\textrm{P,release}}}. \end{aligned}$$

The overall specific product release rate ($\textrm{q}_{\textrm{P,release}})$ was then calculated as the sum of lysis-associated and viable biomass-associated release rates:

10 $$\begin{aligned} \textrm{q}_{\textrm{P,release}}= q_{P,\textrm{rL}} + q_{P,\textrm{rX}}. \end{aligned}$$

### Parameter estimation and uncertainty quantification

Simulations, parameter estimation (PE), and parameter uncertainty quantification were performed as described by [31]. Parameter bounds and detailed information for each estimated parameter are provided in Table A.2. For each experimental design, growth-related parameters were estimated individually for the batch and exponential fed-batch phases.

In the present study, the model-based analysis focused exclusively on the post-induction phase. The considered state variables were biomass, substrate, total product, and extracellular product concentrations. Measurement data from all biological replicates were jointly used for parameter estimation and uncertainty quantification.

The estimated model parameters were $\textrm{q}_{\textrm{d}} ,$ $q_{\textrm{P,release,max}} ,$ and, depending on the model variant, $p_{\textrm{load}} .$ Parameter identification was primarily based on different observables, with $\textrm{q}_{\textrm{d}}$ informed by biomass measurements, production-related parameters by total Fab concentrations, and product release parameters by extracellular Fab concentrations. In experiments where total Fab fragment concentrations were available, the product yield coefficient $Y_{P/S}$ was additionally estimated.

During the bioprocess screening campaign, several cultivations were affected by process limitations or incomplete datasets. To avoid biased parameter estimation, the corresponding data were treated as follows. In cultivations where oxygen limitation occurred approximately 10–15 h after induction in the microbioreactors (MBRs), the model-based analysis was restricted to time intervals without oxygen limitation. This approach was chosen to avoid introducing bias into the estimation of $$\textrm{q}_{\textrm{d}}$$ and $$\textrm{q}_{\textrm{P,release}} . $$ Within these restricted time windows, the maximum product yield was typically not reached. Furthermore, oxygen limitation triggered increased base addition for pH control, resulting in substantial dilution effects. Under these conditions, the product affinity constant ($$K_{P}) $$, representing production capacity, became non-interpretable. Therefore, in experiments where total product concentrations ($$\textrm{Fab}_{\textrm{Total}}) $$ were available, $$K_{P}$$ was fixed to the value corresponding to the highest experimentally observed product yield to avoid constraining estimation of $$Y_{P/S} . $$ In experiments where total product concentrations were not available, $$Y_{P/S}$$ was fixed to its highest experimentally observed value, while $$K_{P}$$ was varied. This approach prevented artificial limitation of the maximum observed time-resolved product release rate due to constrained product formation kinetics. In addition, the modified mechanistic description of product release rendered $$q_{\textrm{P,release,max}}$$ sensitive to high cell lysis rates. In some experiments, insufficient measurements of extracellular product concentrations during the early production phase resulted in increased uncertainty in estimation of this parameter (Supplementary Information 1C). To enable consistent comparison of product release behavior across cultivations, $$q_{\textrm{P,release,max}}$$ was therefore approximated using the maximum observed time-resolved product release rate ($$q^{\max }_{\textrm{P,release}}) $$. Subsequently, the maximum observed time-resolved viable biomass-associated product release rate ($$q^{\max }_{\textrm{P,rX}}) $$ and lysis-associated product release rate ($$q^{\max }_{\textrm{P,rL}}) $$ were also determined. For comparative evaluation, the relative contributions of both release mechanisms to the overall product release rate were analyzed, whereas the corresponding estimated rates are provided in Supplementary Material 1C. The separation of viable biomass-associated ($$\textrm{q}_{\textrm{P,rX}}) $$ and lysis-associated ($$\textrm{q}_{\textrm{P,rL}}) $$ release mechanisms further improved the interpretability of product release dynamics across cultivations.

### Definition of cellular robustness and Pearson correlation analysis

Cellular robustness was derived from the model-estimated cell lysis rate ($\textrm{q}_{\textrm{d}})$ and used as an optimization target throughout this study. To facilitate interpretation of process performance, cellular robustness was defined as the sign-inverted cell lysis rate ($-\textrm{q}_{\textrm{d}})$, such that favorable process outcomes are represented in the same direction across all optimization targets.

The applied process parameters and the corresponding mean values of the optimization targets ($\textrm{Fab}_{\textrm{Total}} ,$ cellular robustness, $\max (\textrm{q}_{\textrm{P}}) ,$ and $\max (\textrm{q}_{\textrm{P,release}})$ were used to calculate Pearson correlation coefficients. The correlation matrix was computed using the pandas package in Python.

## Results

**Fig. 1:**
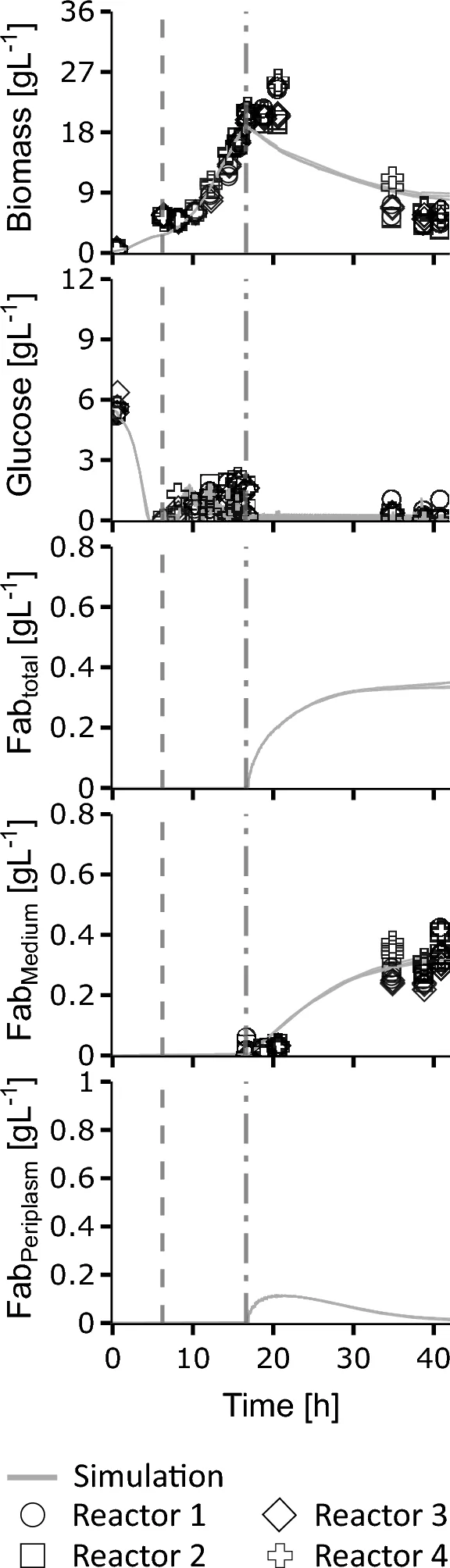
*E. coli* K-12 fed-batch cultivation with Fab production and release under reference conditions. Experimental data (open markers) and predictions of the mechanistic model (solid lines) from Schröder-Kleeberg et al. [25] are shown. The gray vertical dashed and dot–dashed lines indicate the start of feeding and the time of induction, respectively

**Fig. 2:**
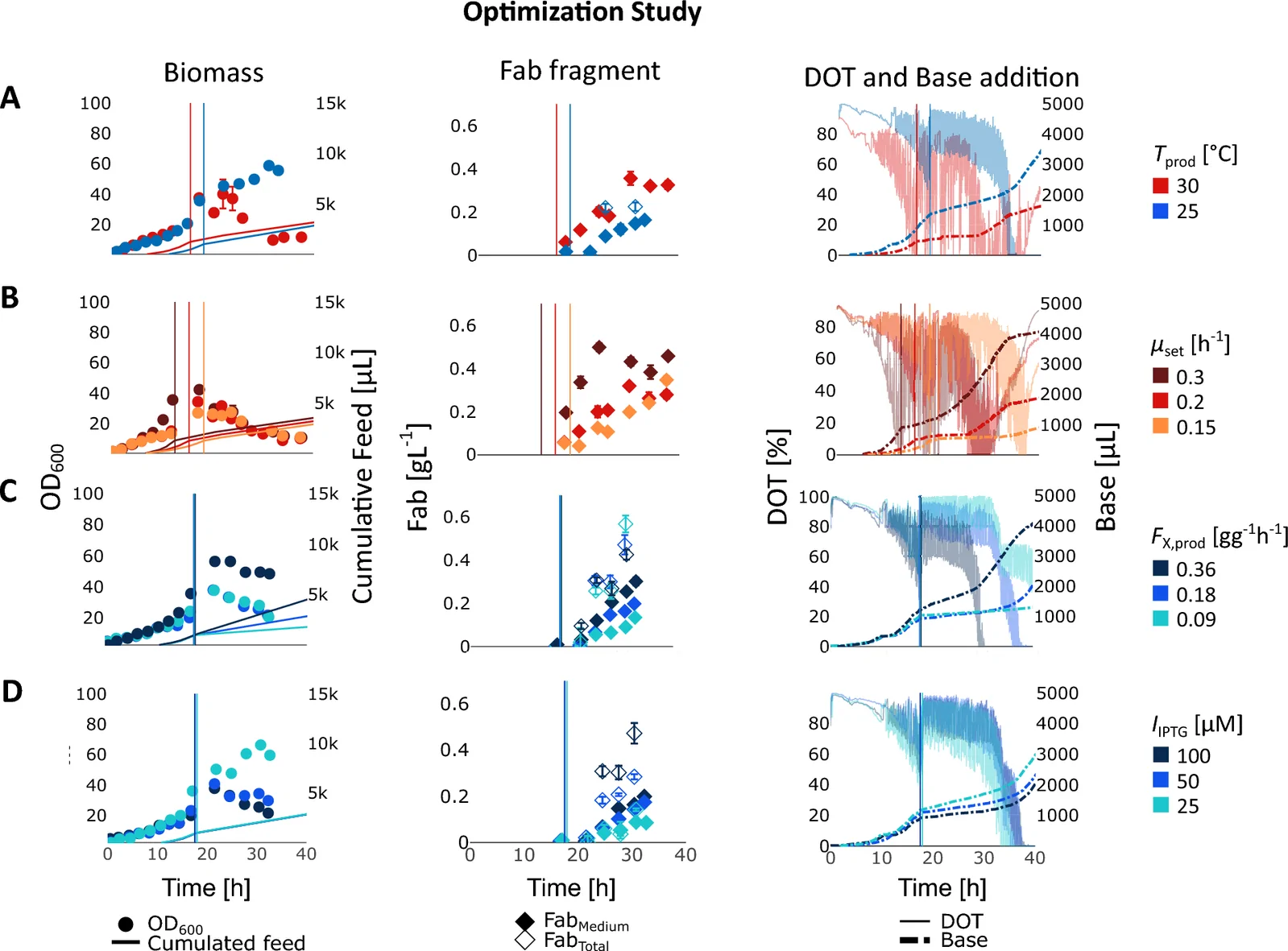
Selected process data from the optimization study. Applied experimental conditions are given as $$T_{\textrm{prod}}/\mu _{\textrm{set}}/F_{\textrm{X,prod}}/I_{\textrm{IPTG}}/F_{\mathrm {\tau }} , $$ corresponding to production temperature ($$^\circ $$C), specific pre-induction growth rate (h$$^{-1}) $$, specific post-induction feed rate (gg$$^{-1}$$h$$^{-1}) $$, induction strength with IPTG (*μ *M), and heterogeneous substrate conditions (min), respectively. Varying conditions are indicated by an “x” (e.g., $$x/\mu _{\textrm{set}}/F_{\textrm{X,prod}}/I_{\textrm{IPTG}}/F_{\mathrm {\tau }}) $$. Shown are the effects of **A** production temperature (reference condition vs. 25/0.2/0.18/50/5), **B** specific growth rate (30/*x*/0.36/100/5), **C** specific post-induction feed rate (25/0.2/*x*/100/5), and **D** induction strength (25/0.2/0.18/*x*/5) on biomass (OD$$_{600}) $$, total product ($$\textrm{Fab}_{\textrm{Total}}) $$, and product in the reactor medium ($$\textrm{Fab}_{\textrm{Medium}}) $$. Additionally, dissolved oxygen tension (DOT, %), cumulative feed, and base addition are plotted. The vertical line indicates the induction time. Reddish data correspond to cultivations at $$T_{\textrm{prod}}= 30~^\circ $$C, while bluish data correspond to $$T_{\textrm{prod}}= 25~^\circ $$C

Bioprocess optimization is inherently multifactorial, requiring evaluation of numerous interacting process parameters to identify conditions that maximize process performance. In addition, optimal operating conditions are often highly specific to the production organism, the target product, and the economic constraints of the process. To identify optimization targets for Fab antibody production in *E. coli*, the bioprocess behavior was first characterized under a defined reference condition, as shown in Fig. 1. The estimated cell dry weight at the time of induction was $20.75 \pm 0.62,\mathrm {g,L^{-1}} ,$ corresponding to approximately $\textrm{OD}_{600} = 50 .$ A key characteristic of the process was pronounced cell lysis, which started immediately after induction and reduced the biomass to approximately 30% of the induction biomass by the end of cultivation. A second characteristic of the process was passive release of the Fab fragment into the reactor medium. Previous work demonstrated that product formation saturates over time [31], which is also reflected in the validation experiment shown in Fig. 4A. Accordingly, the process was designed under the assumption that, after 24.5h of production, most of the accumulated intracellular Fab would be released into the medium, resulting in a final total Fab titer ($\textrm{Fab}_{\textrm{Total}})$ of $0.40 \pm 0.03~\mathrm {g,L^{-1}} .$ The mechanistic model adequately described the reference cultivation (Fig. 1) and enabled estimation of the cell lysis rate ($\textrm{q}_{\textrm{d}})$, product release rate ($\textrm{q}_{\textrm{P,release}})$, and product formation rate ($\textrm{q}_{\textrm{P}})$. The convergence of total and extracellular Fab concentrations toward the end of cultivation suggested that most of the accumulated Fab had been released into the medium, while product formation and product release followed distinct kinetic behaviors. Based on the reference cultivation, three primary optimization targets were identified according to their expected relevance for industrial Fab production: i) maximizing Fab titer, ii) minimizing cell lysis, and iii) improving product release. In addition, the specific product formation rate ($\textrm{q}_{\textrm{P}})$ was included as an auxiliary optimization target because it is mechanistically linked to the three primary objectives.

### Process optimization

The initial yeast extract concentration showed no detectable effect on the optimization targets $\textrm{Fab}_{\textrm{Total}} ,$ $\textrm{q}_{\textrm{d}} ,$ and $\textrm{q}_{\textrm{P,release}}$ under otherwise identical process conditions (e.g., C32 vs. C16). As expected, the readily metabolizable yeast extract-derived nutrients were likely depleted during the batch phase [31]. Consequently, $\textit{YE}_{\textrm{init}}$ was excluded from further analysis.

Accordingly, conditions C14–16 and C30–32 showed nearly identical behavior. C14–16 were retained for further analysis because time-resolved total Fab measurements were available, enabling estimation of $\textrm{q}_{\textrm{P}} .$

Induction strengths of 2 and 10 *μ *M IPTG resulted in no detectable product formation and were excluded from further evaluation. After exclusion of these conditions, 20 combinations of the remaining process parameters were analyzed.

The optimization study was conducted in mini-bioreactors that only support pulse-based feeding and provide limited process control compared with stirred-tank reactors. Therefore, one of the best-performing process conditions was additionally validated in 150 mL stirred-tank reactors (C21).

Figure 2 presents selected process data illustrating the effects of the investigated process parameters on cultivation behavior. However, the displayed time courses do not cover the complete cultivation duration and are therefore not fully representative of the final Fab titers. Under several production conditions, severe foaming occurred during the late production phase, ultimately leading to oxygen limitation in the mini-bioreactors (Fig. 2). Since oxygen limitation substantially affected the optimization targets $\textrm{q}_{\textrm{d}} ,$ $\textrm{q}_{\textrm{P,release}} ,$ and $\textrm{q}_{\textrm{P}} ,$ only data from non-perturbed cultivation phases were considered for comparative analysis and are shown within a common time window. Complete process profilesare provided in Supplementary Information 2, whereas final Fab titers are evaluated separately in conjunction with the model-based analysis.

Cell lysis could be reduced to levels that were no longer reflected by declining biomass concentrations. This effect was primarily achieved by reducing the production temperature to $T_{\textrm{prod}}= 25~^\circ$C (Fig. 2A). In addition, higher post-induction feeding rates (Fig. 2C) and lower induction strengths (Fig. 2D) prevented biomass decline and, in some cases, enabled continued growth after induction. Under these conditions, continuous base addition and sustained biomass accumulation indicated ongoing metabolic activity and suggested that $\mu > \textrm{q}_{\textrm{d}} .$

Reduced production temperatures were associated with substantially slower product release (Fig. 2A). Furthermore, higher post-induction feeding rates and higher pre-induction growth rates ($\mu _{\textrm{set}})$ were generally associated with increased product release (Fig. 2B,C). Higher induction strengths further tended to increase product formation, whereas variations in feeding pulse intervals had no detectable effect on overall process performance (Fig. 4).

Overall, the process data provided a qualitative understanding of how the investigated parameters influenced cell lysis, product formation, and product release. The most prominent observation was that lowering the production temperature substantially reduced cell lysis during production. However, a more quantitative assessment of interactions between process parameters and overall process performance requires additional analytical approaches.

#### Adaptation of the product model

**Fig. 3:**
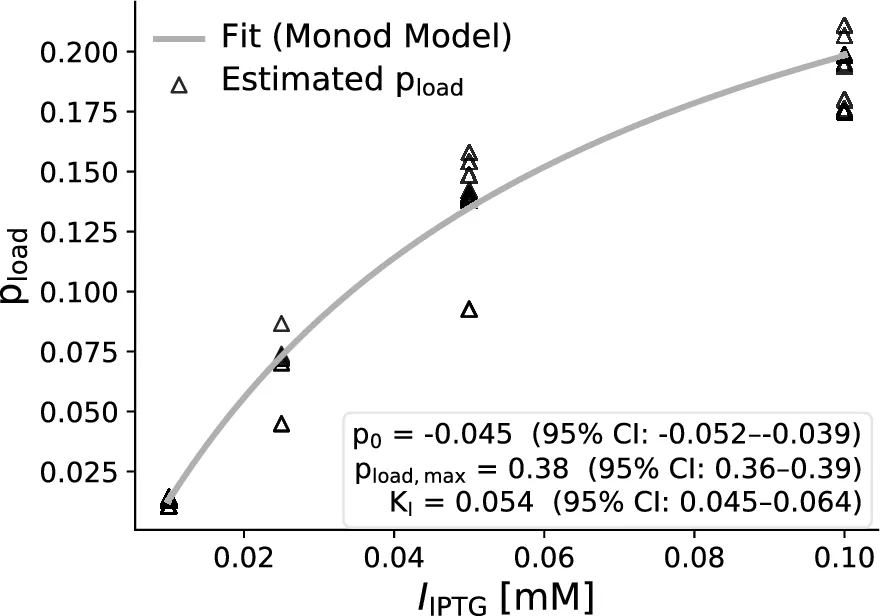
Calibration of the production load $$p_{\textrm{load}}$$ as a function of induction strength with IPTG ($$I_{\textrm{IPTG}}) $$. Values for each $$I_{\textrm{IPTG}}$$ level were obtained using a Monte–Carlo–based method applied to experimental data generated under conditions of high post-induction feed rates. The resulting $$p_{\textrm{load}}$$ estimates were fitted with a Monod-like function to determine the induction offset ($$p_{0}) $$, the maximum production load ($$p_{\textrm{load,max}}) $$, and the affinity to the inducer ($$K_{I}) $$

The product model developed by Schröder-Kleeberg et al. [25] adequately described the extracellular Fab production process under reference conditions (Fig. 1). However, model deviations became apparent at elevated post-induction feeding rates and varying induction strengths (Supplementary Material 3), indicating that product formation and biomass growth could no longer be adequately described. These deviations originated from the assumption that the entire oxidative substrate flux is directed toward product formation after induction ($q_{S,\textrm{ox}} = q_{S,\textrm{ox},P})$. While this assumption remained valid at low feeding rates ($F_{\textrm{X,prod}}= 0.09~\mathrm {g,g^{-1},h^{-1}})$, simultaneous growth and product formation were observed at higher feeding rates and lower induction strengths.

These observations suggested the existence of a finite metabolic production load ($p_{\textrm{load}})$. Additional analyses showed that this parameter was independent of substrate availability (Supplementary Material 3), resulting in the modified formulation given in Eq. 4.

Because $$p_{\textrm{load}}$$ was strongly correlated with the yield coefficient $$Y_{P/S} , $$ independent estimation required conditions under which growth and product formation could be distinguished. Suitable datasets were obtained at $$F_{\textrm{X,prod}}= 0.36~\mathrm {g,g^{-1},h^{-1}}$$ across induction strengths ranging from 2 to 100 *μ *M IPTG.

The estimated production load followed a saturating Monod-like relationship with induction strength and included a non-zero induction offset ($$p_0) $$ (Fig. 3). Parameterization of this relationship enables interpolation and extrapolation of $$p_{\textrm{load}}$$ beyond the experimentally tested induction range, thereby extending the predictive capability of the model.

**Fig. 4:**
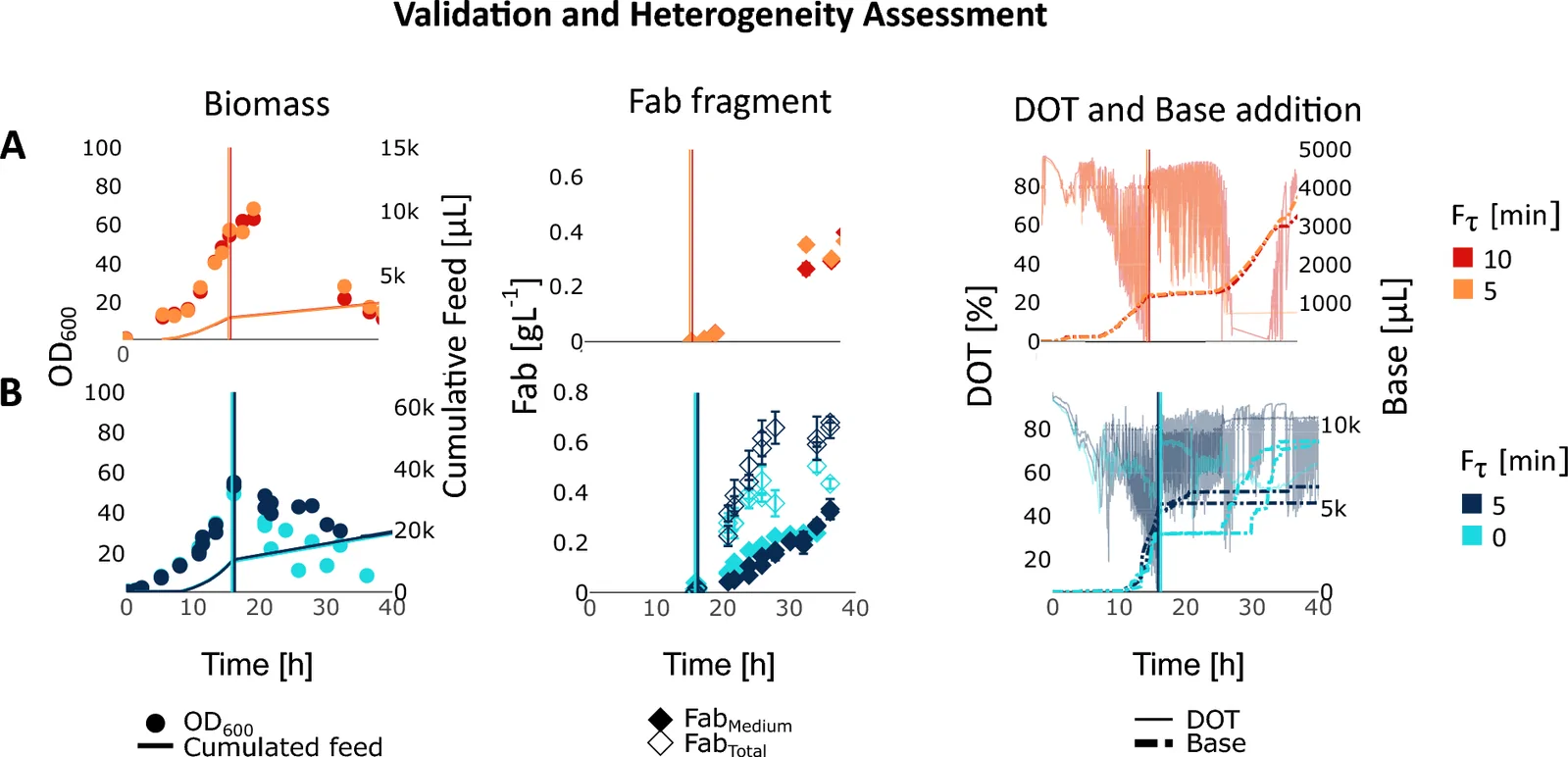
Process data from validation experiments and investigations of robustness against heterogeneous substrate conditions. Applied experimental conditions are given as $$T_{\textrm{prod}}/\mu _{\textrm{set}}/F_{\textrm{X,prod}}/I_{\textrm{IPTG}}/F_{\mathrm {\tau }} , $$ corresponding to production temperature ($$^\circ $$C), specific pre-induction growth rate (h$$^{-1}) $$, specific post-induction feed rate (gg$$^{-1}$$h$$^{-1}) $$, induction strength with IPTG (*μ *M), and heterogeneous substrate conditions (min), respectively. Variable process conditions are indicated by an “x” (e.g., $$x/\mu _{\textrm{set}}/F_{\textrm{X,prod}}/I_{\textrm{IPTG}}/F_{\mathrm {\tau }}) $$. **A** The effect of increased substrate heterogeneity was investigated using larger pulse-based feeding intervals at 15 mL scale ($$30/\text {stepwise feeding}/0.09/100/x) $$. **B** Validation experiments were performed in 150 mL stirred-tank reactors at 25/0.2/0.09/50/5 and compared with continuous feeding (25/0.2/0.09/50/0). Process performance was evaluated based on biomass (OD$$_{600}) $$, total product ($$\textrm{Fab}_{\textrm{Total}}) $$, and product in the reactor medium ($$\textrm{Fab}{\mathrm {_{Medium}}}) $$. Additionally, dissolved oxygen tension (DOT, %), cumulative feed, and base addition are shown. The vertical line indicates the induction time. Reddish data correspond to cultivations at $$T_{\textrm{prod}}= 30~^\circ $$C, whereas bluish data correspond to cultivations at $$T_{\textrm{prod}}= 25~^\circ$$C

#### Model-assisted evaluation of the optimization study

**Fig. 5:**
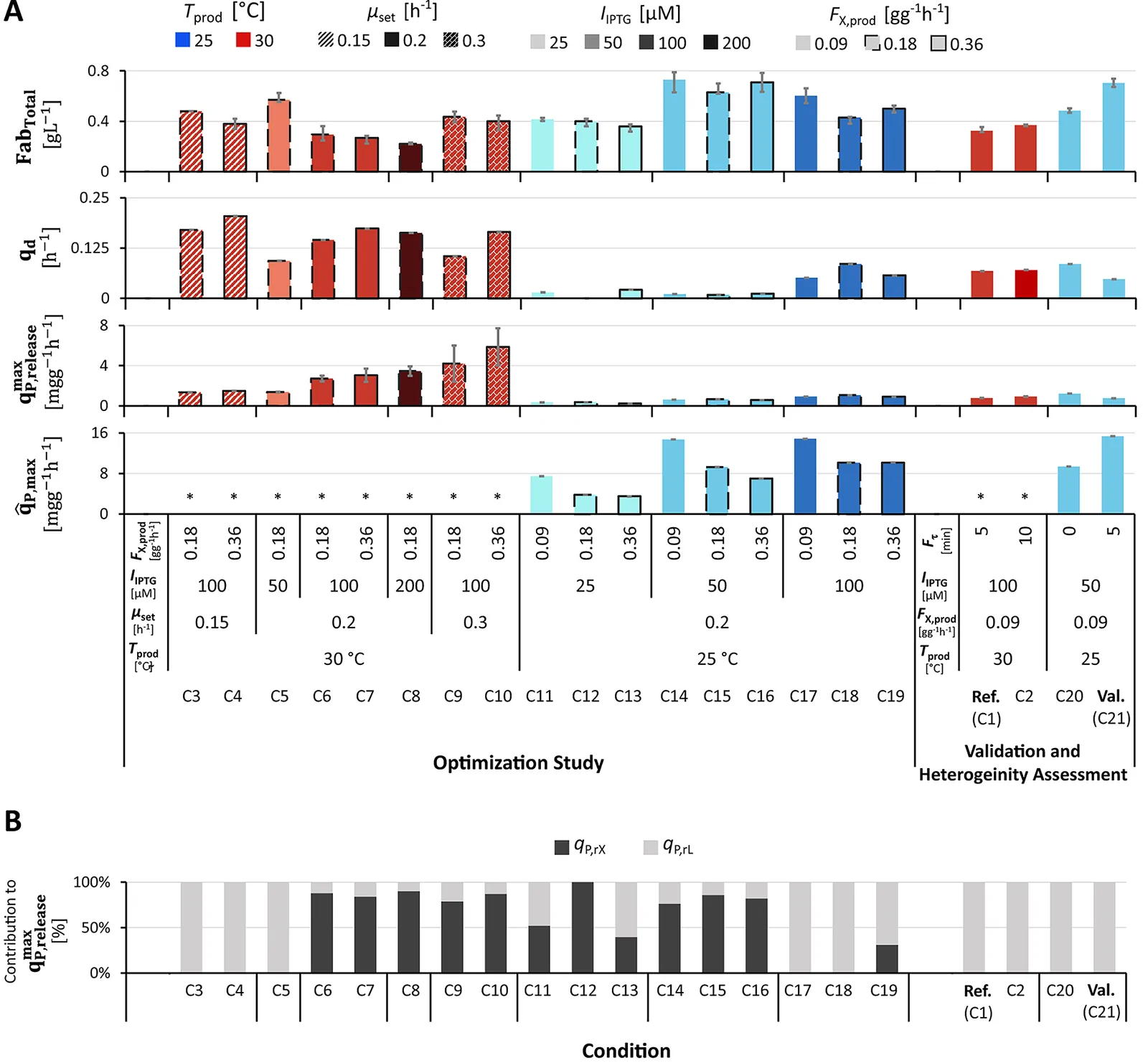
Screening results of an *E. coli*-based Fab antibody production process with product release, evaluated using the optimization targets total Fab titer ($\textrm{Fab}_{\textrm{Total}})$, cell lysis rate ($\textrm{q}_{\textrm{d}})$, maximum specific product release rate ($\textrm{q}_{\textrm{P,release}})$, and maximum specific product formation rate ($\textrm{q}_{\textrm{P}})$. (**A**) Results obtained for 20 process conditions, including the starting condition (reference, Ref.), and compared with an independent validation experiment (Val.). The figure further indicates which conditions were used to investigate substrate heterogeneities through different feeding regimes ($F_{\mathrm {\tau }})$ and which were included exclusively for process optimization. Process conditions are encoded by the post-induction specific feeding rate normalized to the induction biomass ($F_{\textrm{X,prod}} ,$ brightness), the pre-induction specific growth rate ($\mu _{\textrm{set}} ,$ pattern), the production temperature ($T_{\textrm{prod}} ,$ color), and the induction strength ($I_{\textrm{IPTG}} ,$ border style). Conditions marked with an asterisk (*) could not be evaluated. (**B**) Model-based decomposition of the specific product release rate showing the relative contributions of viable biomass-associated product release ($\textrm{q}_{\textrm{P,rX}} ,$ black) and lysis-associated product release ($\textrm{q}_{\textrm{P,rL}} ,$ grey) to total product release for each condition, expressed as percentages

To establish a holistic relationship between the process parameters and the optimization targets, the measured Fab titer ($\textrm{Fab}_{\textrm{Total}})$ and the physiological parameters $\textrm{q}_{\textrm{d}} ,$ $\textrm{q}_{\textrm{P,release}} ,$ and $\textrm{q}_{\textrm{P}}$ were compared across all tested conditions (Fig. 5A). Fab titers were evaluated using the final measured Fab concentrations adjusted for dilution effects. To quantify the underlying cellular dynamics, a model-based approach was applied to estimate the corresponding physiological rates. However, these mechanistic insights could only be derived for the non-perturbed phase of the process, limiting the estimation and interpretation of specific rates (e.g., $\textrm{q}_{\textrm{d}} ,$ $\textrm{q}_{\textrm{P,release}} ,$ and $\textrm{q}_{\textrm{P}})$ to this time window.

**Fig. 6:**
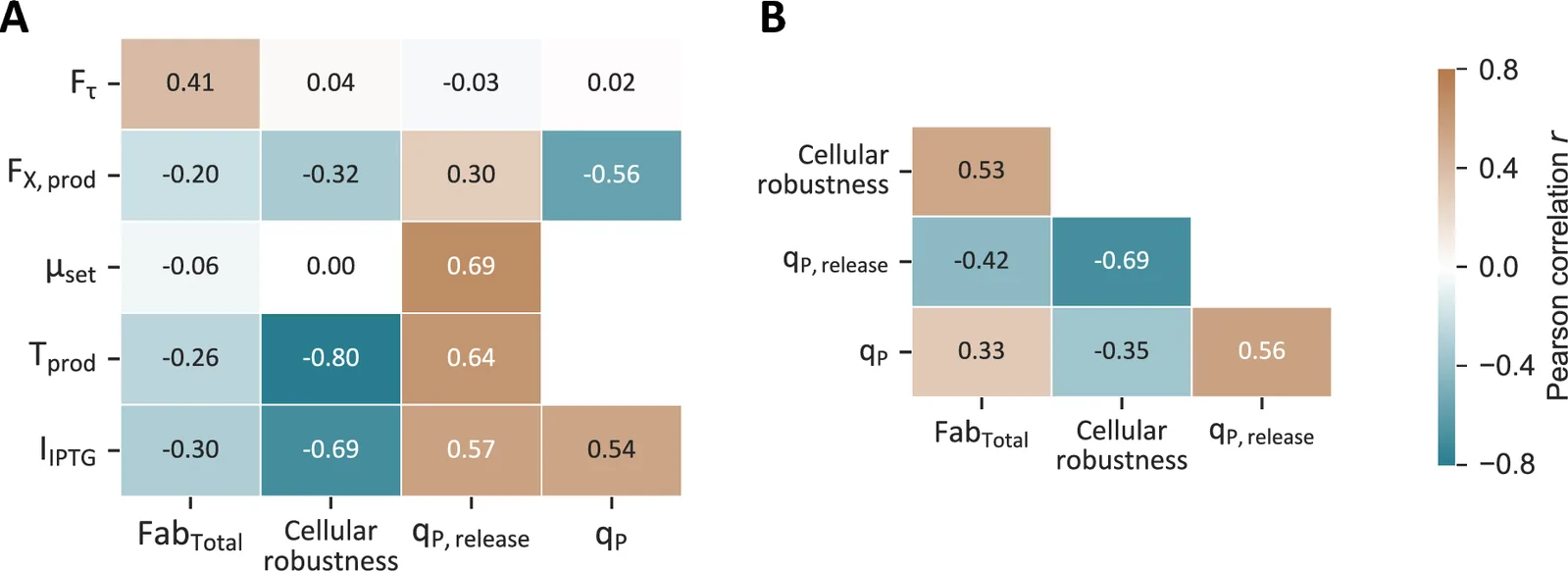
Pearson correlation analysis of the screening results to facilitate identification of key process drivers and trade-offs. Correlations are shown as Pearson correlation coefficients (*r*) and visualized as a heatmap. For improved interpretation, cellular robustness was introduced as a sign-inverted representation of the cell lysis rate ($-\textrm{q}_{\textrm{d}})$, such that favorable process outcomes are represented in the same direction across all optimization targets. (**A**) Effects of the investigated process parameters on the optimization targets. (**B**) Pairwise Pearson correlations between the optimization targets, highlighting synergies and trade-offs between Fab titer, cellular robustness, product formation, and product release

For a reliable interpretation of the estimated parameters, a Monte–Carlo–based method was used to determine parameter uncertainties and thereby assess the identifiability of both the parameters and the model fits. To support interpretation of how the process parameters affect the optimization targets, a Pearson correlation matrix was generated from the data in Fig. 5A and visualized in Fig. 6A. Correlations among the optimization targets themselves were also investigated (Fig. 6B). Cellular robustness was used as optimization target instead of the cell lysis rate to facilitate interpretation of process performance and correlation analyses.

**Product titer.** Following the hierarchy of optimization targets, the highest Fab titer reached approximately $0.7~\mathrm {g,L^{-1}}$ (Fig. 5A, C14 to C16) and was reproduced in the validation experiment (Fig. 5A, C21).

Fab titer was primarily governed by induction strength and production temperature. However, the magnitude of these effects depended strongly on the specific process conditions. At high induction strengths, reducing $T_{\textrm{prod}}$ resulted in up to a 1.5-fold increase in Fab titer (C18), whereas under optimal induction conditions a 1.9-fold increase was observed (C5). The induction strength $I_{\textrm{IPTG}}$ followed a parabolic relationship with Fab titer at both tested production temperatures, with an optimum at *50~μ *M IPTG, enabling up to a twofold increase in yield (C14).

In contrast, $\mu _{\textrm{set}}$ and $F_{\textrm{X,prod}}$ exerted comparatively modest effects on Fab titer (Fig. 5A, $T_{\textrm{prod}}= 30~^\circ$C). Nevertheless, distinct trends were observed for both parameters. The results indicate a non-linear relationship between $\mu _{\textrm{set}}$ and Fab titer, with both low ($\mu _{\textrm{set}}= 0.15)$ and high ($\mu _{\textrm{set}}= 0.3)$ pre-induction growth rates promoting increased Fab titers. Under both growth conditions, lower post-induction feed rates further increased $\textrm{Fab}_{\textrm{Total}} .$

An often overlooked factor in optimization studies is the influence of process heterogeneities. Here, switching from continuous to pulse-based feeding increased Fab titer by approximately 1.4-fold (Fig. 5A, C20 and C21).

Overall, Fab titer was improved up to 2.2-fold relative to the reference process, primarily driven by low $T_{\textrm{prod}}$ and optimal $I_{\textrm{IPTG}} .$ Although the remaining process parameters showed only moderate individual effects, their combined application highlights the potential for further improvements in Fab production.

**Cell lysis and cellular robustness.** To exploit the economic advantages of *E. coli* strains with extracellular product release systems, ideally the entire product should be transferred into the medium without release of additional intracellular components. Minimizing cell lysis is therefore essential for efficient process operation.

Similarly to Fab titer, cellular robustness was primarily governed by production temperature and induction strength. As cellular robustness was derived from the model-estimated cell lysis rate, low $T_{\textrm{prod}}$ and $I_{\textrm{IPTG}}$ corresponded to reduced cell lysis and therefore increased cellular robustness (Fig. 6A), confirming the interpretation of the process data (Fig. 2).

At optimal induction strength, these two parameters alone reduced the cell lysis rate by up to 11.2-fold, reaching a nearly negligible value of $\textrm{q}_{\textrm{d}}= 0.009~\mathrm {h^{-1}}$ (C5 and C15). Validation experiments performed under these conditions showed a higher, although still low, cell lysis rate of $\textrm{q}_{\textrm{d}}= 0.048~\mathrm {h^{-1}} ,$ highlighting the sensitivity of cellular robustness during production.

Switching from pulse-based to continuous feeding at a low feed rate of $F_{\textrm{X,prod}}= 0.09~\mathrm {g,g^{-1},h^{-1}}$ increased the cell lysis rate by approximately 1.8-fold (C20).

Cell lysis rates were further influenced by the specific post-induction feeding rate, although this effect was primarily observed at elevated production temperatures. For example, reducing $F_{\textrm{X,prod}}$ from 0.36 (C7) to 0.18 (C6) decreased the cell lysis rate by approximately 1.4-fold. This trend was consistently observed across different pre-induction growth rates.

In contrast, no clear effect of $\mu _{\textrm{set}}$ on cellular robustness was identified under the tested conditions (Fig. 6A).

Overall, conditions combining $I_{\textrm{IPTG}}= 50~\mu$M, $T_{\textrm{prod}}= 25~^\circ$C, and $F_{\mathrm {\tau }}= 5~\textrm{min}$ resulted in minimal to negligible cell lysis rates while maintaining high Fab titers. Under elevated production temperatures, $\textrm{q}_{\textrm{d}}$ could be further decreased by adjusting $\mu _{\textrm{set}}$ and $F_{\textrm{X,prod}} .$

**Product release.** Consistent with the process data (Fig. 2), the model-based analysis confirmed that elevated production temperatures substantially enhanced product release (Fig. 5, $\textrm{q}_{\textrm{P,release}})$. Under otherwise identical process conditions, increasing $T_{\textrm{prod}}$ from 25 to $30~^\circ$C increased the specific product release rate from $0.58~\mathrm {mg\,g^{-1}\,h^{-1}}$ (C19) to $3.04~\mathrm {mg\,g^{-1}\,h^{-1}}$ (C7), corresponding to an approximately sixfold increase. In addition, applying a higher pre-induction growth rate of $\mu _{\textrm{set}}= 0.3$ further improved product release by up to 1.6-fold.

Product release was frequently accompanied by simultaneous cell lysis, reflected by the strong correlation between both parameters (Fig. 6B). In several cultivations, extracellular product accumulation could be adequately described solely by lysis-associated product release ($\textrm{q}_{\textrm{P,rL}}$; e.g., Fig. 5B, C3). However, 12 process conditions required an additional viable biomass-associated product release term ($\textrm{q}_{\textrm{P,rX}})$ for adequate model description.

At $T_{\textrm{prod}}= 30~^\circ$C, increased $\textrm{q}_{\textrm{P,release}}$ values at elevated $I_{\textrm{IPTG}} ,$ $F_{\textrm{X,prod}} ,$ and $\mu _{\textrm{set}}$ were primarily associated with $\textrm{q}_{\textrm{P,rX}} .$ Under low-temperature conditions, process settings combining reduced induction strengths ($I_{\textrm{IPTG}}\le 50~\mu$M) with improved cellular robustness were also mainly characterized by $\textrm{q}_{\textrm{P,rX}} .$ Under these conditions, the ratio between $\textrm{q}_{\textrm{P,rX}}$ and $\textrm{q}_{\textrm{P,rL}}$ became highly sensitive to the estimated cell lysis rate, as demonstrated by the validation experiment (C21).

Among the remaining process parameters, $F_{\mathrm {\tau }}$ showed no identifiable effect on product release, whereas $F_{\textrm{X,prod}}$ exhibited only a minor influence (Fig. 6A). The observed increase in $\textrm{q}_{\textrm{P,release}}$ at elevated $F_{\textrm{X,prod}}$ is likely linked to enhanced product formation.

**Specific product formation rate.** As shown in Fig. 2D and Fig. 5, the specific product formation rate increased with induction strength following a saturating profile. In addition, $\textrm{q}_{\textrm{P}}$ was influenced by the specific post-induction feeding rate. Across all induction strengths, the highest product formation rates were observed at $F_{\textrm{X,prod}}= 0.09~\mathrm {g,g^{-1},h^{-1}} .$ Increasing the feed rate reduced $\textrm{q}_{\textrm{P}}$ by an average factor of approximately 1.7.

An additional effect was observed for the feeding regime. Switching from continuous to pulse-based feeding increased $\textrm{q}_{\textrm{P}}$ by approximately 1.8-fold.

**Cross-over evaluation.** The results identified $T_{\textrm{prod}}$ and $I_{\textrm{IPTG}}$ as the dominant process parameters across all investigated optimization targets. The optimal induction strength appeared largely independent of production temperature. At this optimal $I_{\textrm{IPTG}} ,$ some of the best performances across all optimization targets were obtained (Fig. 5A, C5 and C14), indicating that induction strength represents a key prerequisite for further process optimization.

Production temperature exerted the strongest influence on cellular performance and revealed a pronounced trade-off between Fab titer, cellular robustness, and product release. Fab titer benefited from high cellular robustness at low $T_{\textrm{prod}} ,$ whereas product release and likely also $\textrm{q}_{\textrm{P}}$ were enhanced at elevated temperatures, which simultaneously increased cell lysis.

In contrast, $\mu _{\textrm{set}}$ and $F_{\textrm{X,prod}}$ exhibited comparatively minor individual effects but shifted the balance of the trade-off toward either improved product release or enhanced cellular robustness. In particular, both parameters improved the viable biomass-associated product release term $\textrm{q}_{\textrm{P,rX}} .$

Finally, pulse-based feeding significantly improved Fab titer, cellular robustness, and $\textrm{q}_{\textrm{P}} ,$ highlighting feeding regime selection as an additional optimization factor for future process development.

## Discussion

This study demonstrates that optimization of extracellular Fab production in *E. coli* requires balancing three competing objectives: maximizing Fab titer, minimizing cell lysis, and promoting product release. Model-assisted analysis revealed that these objectives are strongly interconnected and primarily governed by induction strength and production temperature, which acted as the dominant process drivers. In contrast, $F_{\textrm{X,prod}} ,$ $\mu _{\textrm{set}} ,$ and $F_{\mathrm {\tau }}$ mainly influenced the balance between Fab titer, product release, and cellular robustness. While low production temperatures and moderate induction strengths favored high Fab titers and cellular robustness, elevated temperatures promoted product release but also increased cell lysis. Thus, process optimization in this production system requires not only maximizing product formation but also controlling the physiological trade-off between product release and cellular stability.

### Major optimization factors

Among the investigated process parameters, induction strength ($I_{\textrm{IPTG}})$ exerted one of the strongest influences on all optimization targets. Across the investigated design space, the best overall performances were consistently obtained at the optimal $I_{\textrm{IPTG}} .$ The induction response exhibited a parabolic dependence, with a global optimum at 50 *μ *M IPTG. Lower induction levels were sufficient to activate the promoter at low expression strength (Fig. 5A), maintaining a low metabolic burden and preventing cell lysis. Higher-than-optimal $I_{\textrm{IPTG}}$ increased the product formation rate, but at the cost of a substantial metabolic load [6, 33], ultimately reducing the Fab titer by up to 50%. This suggests that excessive induction may overload the periplasmic folding capacity [34–36] or saturate the Sec-translocon [37], leading to misfolding, aggregation, or degradation of Fab chains. Similar induction-dependent behavior was reported by [19], although without mechanistic explanation. These findings identify induction strength as a prerequisite for balancing Fab production, product release, and cellular robustness.

The most decisive lever for navigating the trade-off is the production temperature ($T_{\textrm{prod}})$. In general, temperature governs microbial activity. Lower $T_{\textrm{prod}}$ slows metabolism, whereas higher $T_{\textrm{prod}}$ accelerates it, for example by affecting biochemical composition [38] and fundamental cellular dynamics such as growth rate [39]. In the present study, $T_{\textrm{prod}}$ also influenced the periplasmic transport pathway in agreement with prior observations [7, 40]. Additionally, higher temperatures presumably increased the risk of Fab misfolding, similar to an overinduction-like response, reducing Fab titer by at least 17% (Fig. 5A, C5). Moreover, Fig. 6B shows that faster product release at high $T_{\textrm{prod}}$ is tightly coupled to an elevated risk of cell lysis. This mechanistically explains the observed trade-off: high $T_{\textrm{prod}}$ increases specific product release ($\textrm{q}_{\textrm{P,release}})$, but simultaneously elevates cell lysis ($\textrm{q}_{\textrm{d}})$, thereby limiting the achievable Fab titer.

### Supportive optimization factors and product release

Compared with induction strength and production temperature, the remaining process parameters exerted more moderate effects and primarily influenced the balance between Fab production, product release, and cellular robustness. In particular, the specific pre-induction growth rate ($\mu _{\textrm{set}})$ and the specific post-induction feeding rate ($F_{\textrm{X,prod}})$ provided additional opportunities to fine-tune process performance.

Higher $\mu _{\textrm{set}}$ improved Fab titer and product release under selected conditions. This effect may result from an increased metabolic capacity of the production host due to elevated ribosome content and transcriptional ortranslational capacity [41, 42]. In addition, growth-rate-dependent changes in outer membrane composition have previously been reported [16, 17]. Such changes may alter membrane permeability and thereby facilitate extracellular product release. Consistent with this interpretation, elevated $\mu _{\textrm{set}}$ increased $\textrm{q}_{\textrm{P,release}}$ without increasing cell lysis. Together with the increased contribution of viable biomass-associated product release ($\textrm{q}_{\textrm{P,rX}})$ observed under these conditions (Fig. 5B), this suggests that higher growth rates may facilitate product release through changes in outer membrane permeability while preserving cellular integrity. Additionally, these effects have the potential to reduce the overall process duration.

The specific post-induction feeding rate ($F_{\textrm{X,prod}})$ also influenced the observed trade-off, although its overall impact was less pronounced than that of $I_{\textrm{IPTG}}$ and $T_{\textrm{prod}} .$ Reduced feeding rates lowered cell lysis, consistent with observations by [7] at elevated production temperatures. A lower $F_{\textrm{X,prod}}$ may reduce the product formation burden, thereby improving folding and transport efficiency while limiting periplasmic product accumulation. At low $T_{\textrm{prod}} ,$ these effects were largely already achieved by reduced temperature, making the additional influence of $F_{\textrm{X,prod}}$ comparatively small and primarily resulting in continued biomass growth (Fig. 2C). The increase in $\textrm{q}_{\textrm{P}}$ observed at reduced $F_{\textrm{X,prod}}$ remains unexpected and requires further investigation.

Additional insight into the release mechanism was obtained by comparing process conditions with similar settings but different production temperatures (e.g., C6 and C18). Under these conditions, elevated temperatures resulted in substantially higher contributions of viable biomass-associated product release ($\textrm{q}_{\textrm{P,rX}})$, indicating that temperature directly enhances the passive release mechanism. This interpretation is consistent with previous reports describing increased extracellular product release at elevated temperatures [7, 43].

Overall, the supportive factors primarily fine-tuned the trade-off between Fab production, product release, and cellular robustness. While $F_{\textrm{X,prod}}$ mainly affected the balance between growth, production burden, and cell lysis, elevated $\mu _{\textrm{set}}$ additionally promoted product release from viable biomass. These observations suggest that supportive factors can complement the dominant effects of induction strength and production temperature by improving extracellular product accumulation without necessarily increasing cell lysis. Under selected conditions, the estimated product release rates substantially exceeded those attributable to lysis-associated release alone, highlighting the potential to selectively enhance non-lytic extracellular product release.

### Process evaluation and study limitations

To assess process robustness and the reliability of the high-throughput screening results, cultivations were exposed to both homogeneous and heterogeneous substrate conditions. Such heterogeneities are common in large-scale bioprocesses and are generally known to negatively affect growth, product titer, and product quality [23, 44, 45]. In contrast, moderate substrate heterogeneities improved Fab titer, cellular robustness, and $\textrm{q}_{\textrm{P}}$ in the present study. In particular, the observed increase in cellular robustness is consistent with the findings of [46].

Continuous feeding, however, resulted in markedly lower Fab titers and higher cell lysis rates than pulse-based feeding. This effect was particularly pronounced at low $F_{\textrm{X,prod}}$ values. A possible explanation is that substrate pulses periodically provide sufficient substrate for both product formation and cellular maintenance, whereas continuous feeding at low $F_{\textrm{X,prod}}$ promotes increasingly severe substrate limitation as the specific substrate uptake rate declines [47]. Nevertheless, further studies are required to validate these findings under continuous feeding conditions and to determine whether the beneficial effects of pulse-based feeding can be transferred to larger scales. These observations further suggest that feeding regime selection represents an additional optimization factor for improving process performance. However, the heterogeneities investigated here were one-dimensional and limited to substrate availability. Additional effects associated with increased mixing times, such as pH and oxygen gradients, were not investigated but are known to influence cellular physiology [48].

The process profiles shown in Fig. 2 indicate that oxygen depletion rapidly destabilizes cells and promotes cell lysis. In numerous mini-bioreactor cultivations, oxygen depletion likely occurred as a consequence of severe foaming induced by cell lysis, which may have affected the final Fab concentration. However, the onset of oxygen depletion consistently coincided with a phase in which product formation had already approached saturation in cultivations without oxygen limitation (Fig. 4B). Comparable observations from additional non-oxygen-limited cultivations of the same production strain at larger scales [25] support the assumption that oxygen depletion had only a limited impact on the observed process trends. This interpretation is further supported by the observation that oxygen depletion predominantly occurred after product formation had already approached saturation.

The consistency of the observed trends across multiple optimization targets, their agreement with established physiological principles, and the coherent physiological interpretation provided by the model-based analysis support the robustness of the main conclusions. Nevertheless, final process decisions and industrial implementation require additional experimental validation. Cellular robustness was inferred from the model-derived cell lysis rate rather than direct viability measurements, and extracellular DNA accumulation as well as product purity were not quantified. These attributes are highly relevant for process development and downstream processing because they directly influence purification efficiency and process complexity. Likewise, validation of the reported Fab titers using analytical methods capable of distinguishing monomeric and multimeric Fab species would further strengthen the interpretation of the measured product concentrations. However, assessment of these attributes was beyond the scope of the present screening study, which focused on identifying process drivers and evaluating their impact on process performance. Furthermore, physiological rates were estimated only for non-perturbed cultivation phases. Consequently, the quantitative interpretation of $\textrm{q}_{\textrm{d}} ,$ $\textrm{q}_{\textrm{P,release}} ,$ and $\textrm{q}_{\textrm{P}}$ remains restricted to cultivation periods unaffected by oxygen limitation. Accordingly, the operating conditions identified in this study should be regarded as promising process candidates rather than fully validated production conditions.

The identified trade-off also suggests a potential strategy for futureprocess intensification. The improved cellular robustness observed at lower production temperatures was associated with reduced secretion-associated stress and a lower risk of cell lysis, which contributed to higher Fab titers. After product formation has approached saturation, cellular resources may become less constrained by product synthesis, potentially allowing increased allocation toward maintenance functions. Consequently, product release could be enhanced in a subsequent release phase by applying temperature shifts and potentially further improved by oxygen shifts [18], while maintaining minimized lysis rates. Previous studies reported suboptimal outcomes for temperature shifts during extracellular production [40]. However, these investigations did not distinguish between production and release phases. The proposed concept therefore represents a promising direction for future process intensification.

## Conclusion

This study demonstrates the value of combining automated high-throughput experimentation with model-assisted analysis for bioprocess development. The investigation of six process parameters across 100 fed-batch cultivations enabled systematic characterization of extracellular Fab production in *E. coli* and would not have been feasible without fully automated high-throughput reactor systems. By integrating mechanistic modeling into the screening workflow, physiological process characteristics such as cell lysis, product formation, and product release kinetics could be estimated, enabling interpretation of process responses beyond experimentally accessible measurements.

The combined experimental and model-based analysis revealed that Fab production with product release is governed by a trade-off between Fab titer, product release, and cellular robustness. Production temperature and induction strength emerged as the dominant determinants of process performance, whereas the pre-induction growth rate, post-induction feeding rate, and feeding regime mainly influenced how this trade-off was balanced. Notably, moderate substrate heterogeneities introduced through the feeding regime improved Fab production and cellular robustness under the investigated conditions.

The extensive dataset further enabled refinement of the previously developed product model and provided new insights into the relationship between product release and cellular robustness. In particular, the large and diverse dataset allowed implementation of an induction-strength-dependent production load, improving the description of product formation across the investigated design space. Furthermore, the model-assisted analysis enabled differentiation between lysis-associated and viable biomass-associated product release, thereby providing a more detailed physiological interpretation of extracellular Fab accumulation and revealing process conditions that promoted extracellular product release beyond what could be explained by cell lysis alone.

More broadly, the study illustrates how mechanistic models can enhance the interpretation of high-throughput screening experiments by linking observable process responses to underlying physiological behavior and by supporting more knowledge-driven optimization decisions. Future work should focus on experimental validation of the most promising process conditions and on further elucidating the mechanisms governing product release. In particular, the observed trade-off suggests that separating product formation and product release into distinct process phases may represent a promising strategy for future process intensification.

## Data Availability

The modeling framework used in this study and the supplementary information are publicly available and can be accessed via the following link: https://git.tu-berlin.de/bvt-htbd/public/schroeder-kleeberg_2024_model_assisted_ht_fab_optimization.

## A Appendix

### A.1 Model Equations

For readers interested in a detailed description of the growth kinetics, we refer to [31], and for a comprehensive explanation of product formation, product release, and lysis behavior, we refer to Schröder-Kleeberg [25].

#### A.1.1 Ordinary equations

11 $$\begin{aligned} \frac{d\textrm{YEF}_{A}}{dt} = -\frac{F}{V}\,\textrm{YEF}_{A} - q_{\textrm{YEF}_{A}}\,X \end{aligned}$$

12 $$\begin{aligned} \frac{d\textrm{YEF}_{B}}{dt} = -\frac{F}{V}\,\textrm{YEF}_{B} - q_{\textrm{YEF}_{B}}\,X \end{aligned}$$

13 $$\begin{aligned} \frac{d\textrm{YE}}{dt} = \frac{d\textrm{YEF}_{A}}{dt} + \frac{d\textrm{YEF}_{B}}{dt} \end{aligned}$$

14 $$\begin{aligned} \frac{dS}{dt} = \frac{F}{V}\,(S_{i} - S) - q_{S}\,X \end{aligned}$$

15 $$\begin{aligned} \frac{dA}{dt} = -\frac{F}{V}\,A + q_{A}\,X \end{aligned}$$

16 $$\begin{aligned} \frac{dP_{\textrm{Fab}}}{dt} = - \frac{F}{V} \, P_{\textrm{Fab,Total}} + \textrm{q}_{\textrm{P}}\, X \end{aligned}$$

17 $$\begin{aligned} \frac{dP_{\textrm{Fab,M}}}{dt} = -\,\frac{F}{V} \, P_{\textrm{Fab,M}} + \textrm{q}_{\textrm{P,release}}\, X \end{aligned}$$

18 $$\begin{aligned} \frac{dP_{\textrm{Fab,PP}}}{dt} = \frac{dP_{\textrm{Fab,Total}}}{dt} - \frac{dP_{\textrm{Fab,M}}}{dt} \end{aligned}$$

19 $$\begin{aligned} \frac{dX}{dt} = -\,\frac{F}{V} \, X + \mu X - \textrm{q}_{\textrm{d}}\, X \, \textrm{event}_{\textrm{Induction}} \end{aligned}$$

20 $$\begin{aligned} \frac{dX_{\textrm{dead}}}{dt} = -\,\frac{F}{V} \, X_{\textrm{dead}} + \textrm{q}_{\textrm{d}}\, X \, \textrm{event}_{\textrm{Induction}}\end{aligned}$$

21 $$\begin{aligned} \frac{d\textrm{DOT}_{m}}{dt} = K_{sensor}\,(\textrm{DOT} - \textrm{DOT}_{m}) \end{aligned}$$

#### A.1.2 Algebraic equations

22 $$\begin{aligned} \textrm{YEF}_{A,0} = \textrm{YE}_{0} \cdot d_{\textrm{YE,AB}}, \end{aligned}$$

23 $$\begin{aligned} \textrm{YEF}_{B,0} = \bigl ( \textrm{YE}_{0} - \textrm{YEF}_{A,0} \bigr ) \cdot d_{\textrm{YE,BC}}\end{aligned}$$

24 $$\begin{aligned} q_{\textrm{YEF}_{A}} = q_{\textrm{YEF}_{A},\max } \,\frac{ \textrm{YEF}_{A}}{\textrm{YEF}_{A} + K_{\textrm{YEF}_{A}}} \end{aligned}$$

25 $$\begin{aligned} q_{\textrm{YEF}_{B}} = q_{\textrm{YEF}_{B},\max } \,\frac{ \textrm{YEF}_{B}}{\textrm{YEF}_{B} + K_{\textrm{YEF}_{B}}} \end{aligned}$$

26 $$\begin{aligned} q_{S} = q_{S,\textrm{max}} \, \frac{S}{S + K_{s}} \, \textrm{TIF} \end{aligned}$$

27 $$\begin{aligned} {\textrm{TIF} = \frac{ \begin{aligned} 1.44 \;+\; (q_{S,\textrm{max}} - q_{S,\textrm{max},T_{\textrm{default}}}) \\ \;-\; 0.186\,T \;+\; 0.00754\,T^{2} \;-\; 6.93\times 10^{-5}\,T^{3} \end{aligned} }{ \begin{aligned} 1.44 \;-\; 0.186\,T_{\textrm{default}} \\ \;+\; 0.00754\,T_{\textrm{default}}^{2} \;-\; 6.93\times 10^{-5}\,T_{\textrm{default}}^{3} \end{aligned} } } \end{aligned}$$

28 $$\begin{aligned} q_{S,\textrm{ox}} = \frac{q_{S}}{1 + \tfrac{q_{\textrm{YEF}_{A}}}{K_{i,qSox,\textrm{YEF}_{A}}} + \tfrac{q_{\textrm{YEF}_{B}}}{K_{i,qSox, \textrm{YEF}_{B}}}}\,\alpha \end{aligned}$$

29 $$\begin{aligned} \alpha = \frac{K_{qSox}}{K_{qSox} + q_{S}} \end{aligned}$$

30 $$\begin{aligned} q_{S,\textrm{ox},P} = q_{S,\textrm{ox}} \, e^{-\,P / K_{P}} \end{aligned}$$

31 $$\begin{aligned} \textrm{q}_{\textrm{P}}= q_{S,\textrm{ox},P} \, Y_{P/S} \end{aligned}$$

32 $$\begin{aligned} q_{m,a} = {\left\{ \begin{array}{ll} q_{m}, & q_{m} \le q_{S,\textrm{ox}} - q_{S,\textrm{ox},P} \\ q_{S,\textrm{ox}} - q_{S,\textrm{ox},P}, & q_{m} > q_{S,\textrm{ox}} - q_{S,\textrm{ox},P} \end{array}\right. } \end{aligned}$$

33 $$\begin{aligned} \textrm{q}_{\textrm{P,release}}= q_{\textrm{P,release,max}} \, \frac{P_{\textrm{Fab,PP}}}{P_{\textrm{Fab,PP}} + K_{\textrm{P,release}}} \end{aligned}$$

34 $$\begin{aligned} q_{S,\textrm{of}} = q_{S} - q_{S,\textrm{ox}} \end{aligned}$$

35 $$\begin{aligned} q_{Ap} = q_{S,\textrm{of}} \cdot Y_{A/S} \end{aligned}$$

36 $$\begin{aligned} q_{Ac} = \tfrac{q_{Ac,\max }}{1 + \tfrac{S}{K_{i,A,S}}}\,\frac{ A }{A + K_{A}} \end{aligned}$$

37 $$\begin{aligned} q_{A} = q_{Ap} - q_{Ac} \end{aligned}$$

38 $$\begin{aligned} {\begin{aligned} \mu =\;&\underbrace{\Bigl ((q_{S,\textrm{ox}} - q_{m})\,Y_{X/S,\textrm{em}}\Bigr )}_{\mu _{S}} \\&+ \underbrace{\Bigl (q_{\textrm{YEF}_{A}}\,Y_{X/\textrm{YEF}_{A}} + q_{\textrm{YEF}_{B}}\,Y_{X/\textrm{YEF}{B}}\Bigr )}_{\mu _{\textrm{YE}}} \\&+ \underbrace{\Bigl (q_{\textrm{Ac}}\,Y_{X/A}\Bigr )}_{\mu _{A}} \end{aligned} } \end{aligned}$$

39 $$\begin{aligned} {\begin{aligned} q_{O} =\;&Y_{O/S}\, \underbrace{\Bigl (q_{S,\textrm{ox}} - \underbrace{\mu _{S}\,\tfrac{C_{X}}{C_{S}}}_{q_{S,\textrm{ox},\mu ,an}}\Bigr )}_{q_{S,\textrm{ox},en}} \\&+ Y_{O/A}\, \underbrace{\Bigl (q_{\textrm{Ac}} - \underbrace{\mu _{A}\,\tfrac{C_{A}}{C_{S}}}_{q_{Ac,\mu ,an}}\Bigr )}_{q_{Ac,en}} \\&+ Y_{O/\textrm{YE}}\, \underbrace{\Bigl ((q_{\textrm{YEF}_{A}} + q_{\textrm{YEF}_{B}}) - \underbrace{\mu _{YE}\,\tfrac{C_{YE}}{C_{S}}}_{q_{YE,\mu ,an}}\Bigr )}_{q_{\textrm{YE},en}} \end{aligned} } \end{aligned}$$

40 $$\begin{aligned} \textrm{DOT} = \textrm{DOT}^{*} - \frac{q_{O}\,X\,H}{k_{L}a} \end{aligned}$$

### A.2 Nomenclature

See appendix Table 2.

**Table 2:** Model state variables and parameters with abbreviations, names, units, and bounds

Abbreviations	Name	Unit	Bound [min,max]
*A*	Acetate	g L$^{-1}$	–
*C*	Carbon content	–	–
*DOT*	Dissolved oxygen tension in equilibrium with the gas phase	% air sat. L g$^{-1}$	–
*S*	Substrate	g L$^{-1}$	–
*YE*	Yeast Extract	g L$^{-1}$	–
$YEF_{i}$	Yeast Extract Fraction *i*, where *i* denotes a specific yeast extract fraction	g L$^{-1}$	–
*X*	Biomass	g L$^{-1}$	–
$K_A$	Affinity constant, acetate consumption	g L$^{-1}$	[0.01, 1.0]
$K_{i,A,S}$	Inhibition constant, inhibition of acetate uptake by substrate	g L$^{-1}$	[0.001, 50]
$K_{i,q_{Sox},\text {YEF}_{A}}$	Inhibition constant, intracellular oxidative substrate uptake by YE fraction A	g L$^{-1}$	[0.001, 100]
$K_{i,q_{Sox},\text {YEF}_{B}}$	Inhibition constant, inhibition of oxidative substrate uptake by YE fraction B	g L$^{-1}$	[0.001, 100]
$K_P$	Affinity constant, product formation	g L$^{-1}$	[0.01, 0.35]
$K_{P,release}$	Affinity constant, product release	g L$^{-1}$	
$k_La$	Volumetric mass transfer coefficient	h$^{-1}$	–
$K_S$	Affinity constant, substrate consumption	g L$^{-1}$	[0.01, 0.09]
$K_{Sensor}$	Dissolved oxygen probe response time	h	
$K_{\text {YEF}_A}$	Affinity constant, consumption of YE fraction A	g L$^{-1}$	[0.01, 1.0]
$K_{\text {YEF}_B}$	Affinity constant, consumption of YE fraction B	g L$^{-1}$	[0.01, 5.0]
$K_{qSox}$	Affinity constant, uptake of substrate for oxidative metabolism	g L$^{-1}$	[0.01, 100]
$p_{load}$	Production load	-	[0, 1]
$q_{Ac,\max }$	Specific acetate consumption rate	g g$^{-1}$ h$^{-1}$	[0.1, 1.5]
$q_{d}$	Lysis rate	h$^{-1}$	[0, 0.5]
$q_{P,release,\max }$	Maximum specific product release rate	g g$^{-1}$ h$^{-1}$	[0, 0.03]
$q_{m}$	Specific maintenance rate	g g$^{-1}$ h$^{-1}$	–
$q_{S,\max }$	Maximum specific substrate uptake rate	g g$^{-1}$ h$^{-1}$	[0.2, 1.5]
$q_{\text {YEF}_A,\max }$	Maximum specific uptake rate for YE fraction A	g g$^{-1}$ h$^{-1}$	[0.5, 3.0]
$q_{\text {YEF}_B,\max }$	Maximum specific uptake rate for YE fraction B	g g$^{-1}$ h$^{-1}$	[0.1, 2.0]
*TIF*	Temperature influencing factor for $q_S$	-	
$Y_{A/S}$	Yield of acetate per gram of substrate	g g$^{-1}$	–
$Y_{O/A}$	Oxygen used per gram of acetate metabolized	g g$^{-1}$	–
$Y_{P/S}$	Yield of product per gram of substrate	g g$^{-1}$	[0, 0.3]
$Y_{X/S,em}$	Yield of biomass per gram of substrate (glucose) exclusive maintenance	g g$^{-1}$	[0.3, 0.5]
$Y_{X/\text {YEF}_A}$	Yield of biomass per gram of YE fraction A	g g$^{-1}$	[0.2, 1.0]
$Y_{X/\text {YEF}_B}$	Yield of biomass per gram of YE fraction B	g g$^{-1}$	[0.05, 0.2]
$Y_{X/A}$	Yield of biomass per gram of acetate	g g$^{-1}$	[0.25, 0.45]
$Y_{O/S}$	Oxygen used per gram of substrate metabolized	g g$^{-1}$	–
$Y_{O/YE}$	Oxygen used per gram of YE metabolized	g g$^{-1}$	–
